# Computational Modeling of Oxygen Delivery in Norwood Physiology: Differential Effects of Systemic and Pulmonary Vasodilator Conditions

**DOI:** 10.3390/jcdd13080347

**Published:** 2026-07-23

**Authors:** Fabio Savorgnan, Vikram Shah, E’Kiijah Turner, Kathryn Hu, Pranathi Pilla, Sarah Visokay, Kaitlin Ness, Saul Flores, Rohit Loomba, Sebastian Acosta

**Affiliations:** 1 Department of Pediatrics, Division of Critical Care, Baylor College of Medicine, Houston, TX 77030, USA; pranathi.pilla@bcm.edu (P.P.);; 2 Department of Computational Applied Mathematics and Operations Research, Rice University, Houston, TX 77005, USAkh150@rice.edu (K.H.); 3 Department of Pediatrics, Division of Cardiology, Northwestern University, Evanston, IL 60611, USA; rohit.loomba@northwestern.edu

**Keywords:** Norwood procedure, hypoplastic left heart syndrome, Qp:Qs, oxygen delivery, systemic blood flow, pulmonary blood flow, shunt resistance, pulmonary vascular resistance, systemic vascular resistance, Monte Carlo simulation

## Abstract

Background: After the Norwood operation, systemic and pulmonary circulations are supplied in parallel by a single right ventricle. Pulmonary blood flow depends on both native pulmonary vascular resistance and shunt/conduit resistance; therefore, balanced Qp:Qs can occur despite low native pulmonary resistance. Changes in systemic, pulmonary, or shunt/conduit resistance may alter flow distribution, systemic venous saturation, and oxygen delivery. Objective: To evaluate the predicted hemodynamic and oxygen delivery effects of systemic and pulmonary vasodilator conditions in a computational Norwood circulation model, and to assess the robustness of these effects using Monte Carlo simulation, dose–response analysis, nonlinear shunt modeling, afterload–responsive flow, and Rp/R-shunt sensitivity analysis. Methods: A lumped-parameter Norwood circulation model was used with a balanced baseline state: Qs = Qp = 1.0 L/min, total right ventricular flow = 2.0 L/min, and Qp:Qs = 1:1. The primary steady-state fixed-flow model was supplemented with sensitivity analyses incorporating afterload–responsive ventricular output, nonlinear shunt/conduit resistance, dose–response simulations, and a two-parameter Rp/R-shunt oxygen delivery surface. Results: In the fixed-flow model, nicardipine increased systemic flow and improved oxygen delivery, whereas pulmonary vasodilators shifted flow toward the pulmonary circulation and could reduce systemic flow. In the extended sensitivity analyses, predicted drug effects varied with ventricular flow reserve and the relationship between native pulmonary resistance and shunt/conduit resistance. Conclusions: In this Norwood circulation model, oxygen delivery depended primarily on systemic flow, ventricular response to afterload reduction, and the Rp/R-shunt relationship. Pulmonary vasodilators may improve modeled saturation but can reduce oxygen delivery when pulmonary runoff occurs without a compensatory increase in ventricular output.

## 1. Introduction

Hypoplastic left heart syndrome and related single-ventricle lesions require staged surgical palliation beginning with the Norwood operation [1,2,3]. After Norwood palliation, the right ventricle becomes the effective systemic ventricle and supplies both systemic and pulmonary circulations through a common arterial pathway. Unlike normal biventricular physiology, in which pulmonary and systemic blood flow are arranged in series, the Norwood circulation is arranged in parallel. As a result, pulmonary and systemic circulations compete for the same ventricular output.

This physiology makes Qp:Qs balance central to postoperative management. An intervention that increases pulmonary blood flow may simultaneously reduce systemic blood flow. Therefore, arterial oxygen saturation alone may be misleading: a higher saturation can reflect increased pulmonary blood flow while systemic perfusion and oxygen delivery decline. Naito and colleagues described optimization of systemic oxygen delivery as a major physiologic goal after the Norwood procedure [4]. Li and colleagues showed that oxygen delivery after Norwood palliation was closely related to systemic vascular resistance and hemoglobin, but not to Qp alone [5].

Several studies have examined the relationship between Qp:Qs and oxygen delivery. Barnea and colleagues demonstrated theoretically that systemic oxygen availability depends on the interaction between Qp and Qs rather than pulmonary flow alone [6]. Charpie and colleagues reported that maximal oxygen delivery may occur at Qp:Qs at a rate less than 1 [7]. Photiadis and colleagues similarly found that maximum oxygen delivery occurs at Qp:Qs at a rate less than 1, although broader hemodynamic status may be optimized when Qp:Qs is between 1 and 2 [8].

Systemic venous saturation provides additional insight into the balance between oxygen delivery and oxygen consumption. Hoffman and colleagues found that lower postoperative systemic venous oxygen saturation after Norwood palliation was associated with abnormal childhood neurodevelopmental outcomes [9]. Dhillon and colleagues reported that routine hemodynamic parameters did not accurately reflect oxygen transport after the Norwood procedure, except for systemic venous oxygen saturation [10]. Together, these studies support integrating arterial saturation with Qs, Qp:Qs, systemic venous saturation, lactate, perfusion pressure, and oxygen delivery.

The objective of this study was to evaluate the predicted hemodynamic and oxygen delivery effects of systemic and pulmonary vasodilator conditions in a computational Norwood circulation model, and to assess the robustness of these effects using Monte Carlo simulation, dose–response analysis, nonlinear shunt modeling, afterload–responsive flow, and Rp/R-shunt sensitivity analysis.

## 2. Materials and Methods

### 2.1. Model Structure

A steady-state lumped-parameter model of Norwood physiology was used. The model represents the right ventricle as a single pump supplying a common arterial compartment, from which flow divides into systemic flow, Qs, and pulmonary flow, Qp. Flow distribution is determined by systemic vascular resistance, native pulmonary vascular resistance, and shunt/conduit resistance.

Native pulmonary vascular resistance was modeled as lower than systemic vascular resistance, consistent with postnatal pulmonary vascular transition. Balanced Qp:Qs was achieved by including shunt/conduit resistance in series with the pulmonary vascular bed. Thus, the effective pulmonary pathway resistance was defined as follows:

$$R_{p,eff}=R_{p}+R_{shunt}$$

where $R_{p}$ is native pulmonary vascular resistance, and $R_{shunt}$ is shunt/conduit resistance.

The primary pressure–flow relationships were:

$$Q_{s}=\frac{P_{a}-P_{v}}{R_{s}}\;Q_{p}=\frac{P_{a}-P_{pv}}{R_{p}+R_{shunt}}$$

where $P_{a}$ is arterial pressure, $P_{v}$ is systemic venous pressure, $P_{pv}$ is pulmonary venous/common atrial downstream pressure in the simplified model, $R_{s}$ is systemic vascular resistance, $R_{p}$ is native pulmonary vascular resistance, and $R_{shunt}$ is shunt/conduit resistance.

Total right ventricular flow was defined as follows:

$$Q_{RV}=Q_{s}+Q_{p}$$

This structure reflects Norwood physiology, in which pulmonary blood flow depends on native pulmonary vascular resistance, shunt or conduit resistance, systemic vascular resistance, and shared single-ventricle output [11,12,13].

### 2.2. Baseline Resistance and Compliance Assumptions

The baseline simulation used Qs = 1.00 L/min, Qp = 1.00 L/min, total right ventricular flow = 2.00 L/min, and Qp:Qs = 1.00. To generate this balanced state while preserving lower native pulmonary resistance, baseline systemic resistance was set to approximately 40 mmHg/L/min, native pulmonary resistance to approximately 12 mmHg/L/min, and shunt/conduit resistance to approximately 28 mmHg/L/min. Therefore,

$$R_{s}=40\;\mathrm{mmHg}/L/\min\;R_{p}=12\;\mathrm{mmHg}/L/\min\;R_{shunt}=28\;\mathrm{mmHg}/L/\min\;R_{p,eff}=R_{p}+R_{shunt}=40\;\mathrm{mmHg}/L/\min$$

This allowed the effective pulmonary pathway resistance to approximate systemic resistance and produce Qp:Qs of 1:1. These values were used as baseline simulation assumptions rather than universal normal values for postoperative Norwood patients. This approach is consistent with neonatal physiology showing a fall in pulmonary vascular resistance after birth and with Norwood modeling studies demonstrating that shunt size, shunt resistance, systemic vascular resistance, and pulmonary vascular resistance influence flow distribution and oxygenation [11,12,13].

For clarity, the resistance values used in the baseline model were not intended to represent fixed universal postoperative Norwood values. Rather, they were selected to construct a physiologically plausible balanced state for the simulations. In the primary analysis, systemic resistance and shunt/conduit resistance were held constant except for the specified drug-related resistance changes, whereas the revised sensitivity analyses explicitly tested how changes in the native pulmonary resistance to shunt/conduit resistance relationship modify oxygen delivery.

Ventricular diastolic compliance was held constant at 2–4 mL/mmHg. This range was selected as a simplified neonatal single-ventricle modeling assumption to avoid confounding vascular resistance effects with ventricular dysfunction or altered ventricular–arterial coupling. Lumped-parameter congenital heart disease models commonly use simplified compliance and elastance terms when patient-specific ventricular mechanics are unavailable [13].

### 2.3. Oxygen Transport Framework

The model assumes complete mixing of systemic venous and pulmonary venous blood:

$$S_{mixed}=\frac{S_{SV}Q_{s}+S_{PV}Q_{p}}{Q_{s}+Q_{p}}$$

Systemic venous saturation is determined by oxygen consumption, hemoglobin concentration, systemic flow, and the arteriovenous saturation difference as follows:

$$\dot{V}O_{2}=1.34\times Hb\times (S_{mixed}-S_{SV})Q_{s}$$

Systemic oxygen delivery was conceptualized as follows:

$$DO_{2}=Q_{s}\times C_{a}O_{2}$$

Because hemoglobin and oxygen-carrying capacity were held constant, oxygen delivery was summarized using a simplified oxygen delivery index as follows:

$$DO_{2}index=Q_{s}\times S_{mixed}$$

This index was used to compare the direction of change across drug conditions when systemic flow and saturation moved in opposite directions.

### 2.4. Baseline State and Drug Conditions

Baseline was defined as Qs = 1.00 L/min, Qp = 1.00 L/min, total right ventricular flow = 2.00 L/min, Qp:Qs = 1.00, mixed saturation = 0.80, systemic venous saturation = 0.61, and pulmonary venous saturation = 0.99. This represents a balanced Norwood circulation in which effective pulmonary pathway resistance approximates systemic resistance because of shunt/conduit resistance.

Drug effects were represented as changes in systemic vascular resistance, native pulmonary vascular resistance, or both. Shunt/conduit resistance was held constant across drug conditions because the objective was to isolate the effect of vascular resistance changes rather than shunt size or conduit geometry. After each resistance change, Qs, Qp, Qp:Qs, mixed saturation, systemic venous saturation, and oxygen delivery index were recalculated.

### 2.5. Resistance-Change Assumptions

For the deterministic simulations, each drug condition was represented as a predefined percent change in $R_{s}$, $R_{p}$, or both. Nicardipine was modeled as predominant systemic vasodilation, with $R_{s}$ reduced by 30% and $R_{p}$ reduced by 5%, consistent with its systemic arterial vasodilatory profile [14]. Milrinone was modeled as mixed systemic and pulmonary vasodilation, with $R_{s}$ reduced by 18% and $R_{p}$ reduced by 22.5%, reflecting its inodilator effects and prior neonatal and pediatric cardiac surgery data showing reductions in systemic and pulmonary vascular tone [15,16]. Sildenafil was modeled as preferential pulmonary vasodilation, with $R_{s}\;$ reduced by 10% and $R_{p}$ reduced by 30%, consistent with its pulmonary vascular effects in pediatric pulmonary hypertension, congenital heart disease, and single-ventricle physiology [17,18,19]. Inhaled nitric oxide was modeled as selective pulmonary vasodilation, with no change in $R_{s}$ and a 45% reduction in $R_{p}$, reflecting its selective pulmonary action and rapid inactivation by hemoglobin [20,21]. Epoprostenol was modeled as strong pulmonary vasodilation with systemic vasodilatory effect, with $R_{s}$ reduced by 15% and $R_{p}$ reduced by 40%, consistent with prostacyclin-mediated pulmonary and systemic vasodilation [22,23,24].

These values were selected as physiologically plausible modeling assumptions to compare directional effects across drug conditions. They should not be interpreted as validated drug-specific pharmacodynamic estimates in postoperative Norwood patients.

### 2.6. Monte Carlo Sensitivity Analysis

A 20,000-iteration Monte Carlo sensitivity analysis was performed. Baseline total right ventricular flow was sampled around 2.0 L/min with approximately 10% variability, and baseline Qp:Qs was sampled around 1.0 with approximately 0.10 absolute variability. Drug-induced changes in systemic and native pulmonary vascular resistance were varied around the modeled values by approximately 5 absolute percentage points. Shunt/conduit resistance was held constant within each iteration unless baseline Qp:Qs sampling required adjustment of effective pulmonary pathway resistance. Pulmonary venous saturation was held at 0.99, and the oxygen consumption term was held constant from baseline.

For each iteration, Qs, Qp, and Qp:Qs, mixed saturation, systemic venous saturation, oxygen delivery index, and percent change in oxygen delivery index were recalculated. This analysis was intended to test directional robustness rather than generate patient-specific pharmacodynamic predictions.

### 2.7. Code Implementation

The simulation was implemented in Python (version 3.8) using predefined baseline parameters, drug-specific resistance multipliers, and reusable calculation functions. Baseline systemic resistance, native pulmonary resistance, and shunt/conduit resistance were stored separately. Each drug condition was stored as a dictionary containing the percent change in systemic and native pulmonary vascular resistance. The code applied these resistance multipliers, recalculated effective pulmonary pathway resistance, flow partitioning, and oxygen delivery outputs. The Monte Carlo analysis was implemented with NumPy-based random sampling and iterative recalculation of model outputs, summarized as means, 95% uncertainty intervals, percent change from baseline, and probability of oxygen delivery index decrease.

### 2.8. Advanced Sensitivity Extensions

Additional model extensions were implemented to test whether the primary findings were sensitive to assumptions regarding fixed ventricular output, linear shunt resistance, single-point drug effects, and the relationship between native pulmonary resistance and shunt/conduit resistance.

First, total right ventricular output was allowed to vary with effective parallel afterload using a simplified pressure–flow reserve relationship:

Q-total = Q-total, baseline × (R-afterload, baseline/R-afterload, current)^α

where α is an adjustable afterload-sensitivity parameter. The default value was α = 0.35. This approach was not intended to replace a full time-varying elastance model, but rather to provide a transparent first-order approximation of afterload-dependent changes in ventricular output.

Second, shunt/conduit resistance was modeled with both linear and quadratic components:

ΔP-shunt = K1Q + K2Q^2^

The nonlinear fraction determined how much of the nominal shunt resistance was assigned to the quadratic term. A value of 0 reproduced the original linear Ohmic shunt model, whereas higher values progressively increased nonlinear flow limitation at higher shunt flow.

Third, drug effects were simulated across a dose–response continuum rather than as single predefined percentage changes. For each drug, the modeled maximum changes in systemic and native pulmonary vascular resistance were scaled from 0 to 1.25 times the original assumed effect. Finally, oxygen delivery was mapped across simultaneous variation in native pulmonary resistance and shunt/conduit resistance to identify regions where pulmonary vasodilation was predicted to be beneficial, neutral, or harmful.

## 3. Results

Starting from Qs = Qp = 1.00 L/min and total flow of 2.0 L/min, the modeled drug conditions produced distinct shifts in flow distribution and oxygen delivery. Nicardipine shifted flow toward the systemic circulation, increasing Qs to 1.15 L/min while reducing Qp to 0.85 L/min. Although mixed saturation decreased from 0.800 to 0.769, oxygen delivery index increased from 0.800 to 0.882, a 10.3% improvement (Table 1).

Milrinone produced the most balanced response, with Qs of 0.97 L/min, Qp of 1.03 L/min, Qp:Qs of 1.06, and a small oxygen delivery index decrease of 2.2%. In contrast, sildenafil, inhaled nitric oxide, and epoprostenol shifted flow toward the pulmonary circulation. Sildenafil increased Qp:Qs to 1.29 and reduced oxygen delivery index by 10.2%. Inhaled nitric oxide produced the largest pulmonary shift, increasing Qp:Qs to 1.82 and reducing oxygen delivery index by 25.2%. Epoprostenol increased Qp:Qs to 1.42 and reduced oxygen delivery index by 14.4%. In all three pulmonary vasodilator conditions, modeled mixed saturation increased while systemic flow and oxygen delivery index decreased.

**Figure 1:**
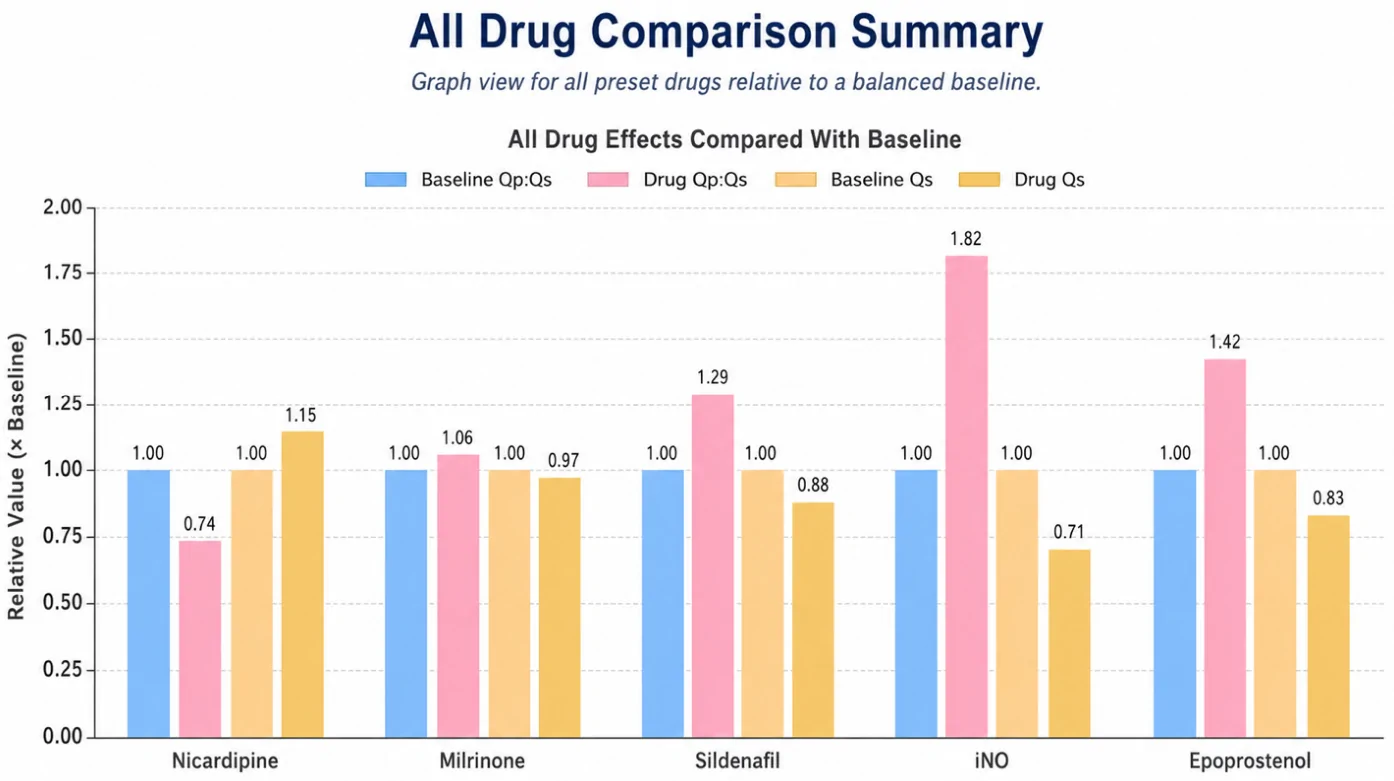
All drug effects compared with the baseline.

The Monte Carlo analysis supported the deterministic findings. Nicardipine consistently improved oxygen delivery index, with a mean increase of 9.9% and 0.0% probability of oxygen delivery index decreasing. Milrinone remained closest to neutral, with a mean oxygen delivery index change of −2.2%; because the effect size was small, its direction was more sensitive to uncertainty than the other drug conditions. Sildenafil, inhaled nitric oxide, and epoprostenol consistently reduced oxygen delivery index despite increasing modeled saturation. The probability of oxygen delivery index decreasing was 99.8% for sildenafil and 100% for both inhaled nitric oxide and epoprostenol. Overall, the direction of effect remained stable across plausible uncertainty in baseline physiology and drug-related resistance changes (Table 2, Figure 1).

### Advanced Sensitivity Analyses

When the simplified afterload–responsive total flow relationship was activated, modeled total ventricular output increased after reductions in effective parallel afterload. Under the default advanced assumptions (afterload sensitivity α = 0.35; nonlinear shunt fraction = 0.5), nicardipine increased total flow from 2.00 to 2.15 L/min and increased oxygen delivery by 20.7%. Milrinone increased oxygen delivery by 10.9%, sildenafil by 5.2%, and epoprostenol by 8.3%, whereas inhaled nitric oxide remained slightly unfavorable, with a 1.3% reduction in oxygen delivery. These findings suggest that the fixed-flow model may underestimate the potential benefit of systemic afterload reduction. They also show that the predicted effects of pulmonary vasodilators depend strongly on whether total ventricular output can increase enough to offset pulmonary runoff.

The nonlinear shunt model also changed the magnitude of pulmonary steal. Increasing the quadratic component of shunt resistance reduced the rise in Qp:Qs during selective pulmonary vasodilation because higher pulmonary flow encountered progressively greater shunt-related pressure loss. Thus, the original fixed linear shunt model should be interpreted as a simplifying assumption rather than a complete description of BT or Sano shunt hemodynamics (Table 3).

**Figure 2:**
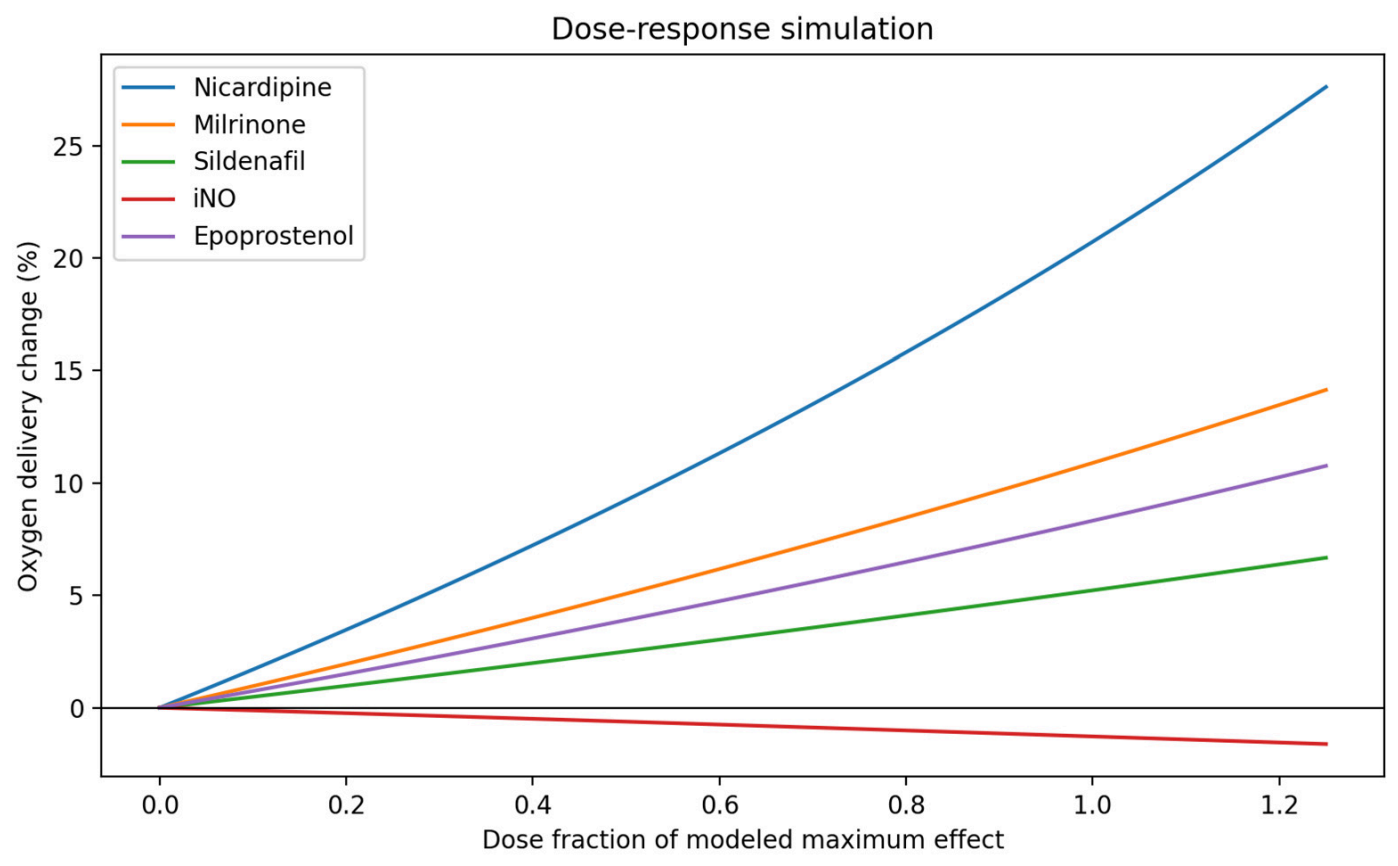
Dose–response simulation across scaled drug effect fractions. The plot shows percent change in oxygen delivery relative to baseline under the advanced sensitivity assumptions.

Advanced sensitivity analysis used alpha = 0.35 for afterload responsiveness and nonlinear shunt fraction = 0.5. Values are model outputs and should not be interpreted as validated clinical pharmacodynamic dose predictions (Figure 2).

**Figure 3:**
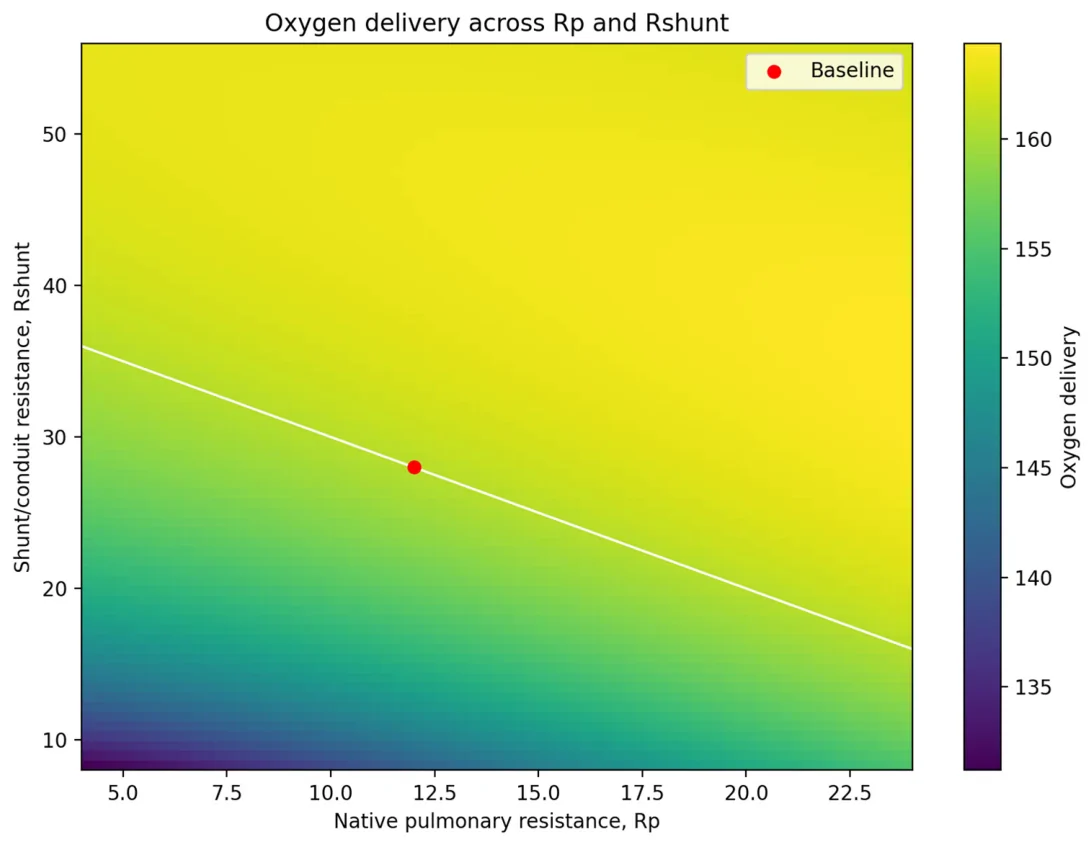
Oxygen delivery surface across native pulmonary resistance and shunt/conduit resistance. The red point marks the baseline assumption Rp = 12 and R-shunt = 28. The white contour marks the baseline oxygen delivery level.

This dose–response simulation shows that the predicted effect on oxygen delivery varies across the modeled drug-effect range rather than behaving as a simple on/off response (Figure 3). Nicardipine produced the largest dose-dependent increase in oxygen delivery, followed by milrinone and epoprostenol, while sildenafil showed a smaller positive effect. In contrast, inhaled nitric oxide produced a slight progressive reduction in oxygen delivery, suggesting that selective pulmonary vasodilation may increase pulmonary runoff without sufficiently improving systemic flow. Overall, the figure supports the concept that vasoactive effects in Norwood physiology are highly dose- and physiology-dependent.

The Rp/R-shunt heatmap demonstrates that oxygen delivery depends on the combined resistance of the native pulmonary vascular bed and the shunt/conduit. At low shunt/conduit resistance, pulmonary runoff can lower systemic flow and oxygen delivery despite improved modeled oxygen saturation. Conversely, higher shunt/conduit resistance limits pulmonary runoff and preserves systemic delivery. This supports the fact that the Rp/R-shunt relationship represents a clinically important threshold for interpreting pulmonary vasodilator risk.

## 4. Discussion

This model demonstrates that, in balanced Norwood physiology, systemic oxygen delivery is highly dependent on preservation of systemic blood flow. The updated model explicitly separates native pulmonary vascular resistance from shunt/conduit resistance. This distinction is important because native pulmonary resistance after birth is expected to be lower than systemic resistance, while the shunt or conduit provides additional resistance that limits pulmonary runoff and allows Qp:Qs to approximate 1:1.

Nicardipine improved the oxygen delivery index by shifting flow toward the systemic circulation, even though modeled saturation decreased modestly. In contrast, sildenafil, inhaled nitric oxide, and epoprostenol increased pulmonary flow and modeled saturation but reduced systemic flow and oxygen delivery index. Milrinone had the most neutral modeled effect under the resistance-only assumptions of the model.

These findings are consistent with the prior Norwood physiology literature. Naito and colleagues emphasized systemic oxygen delivery as a key physiologic goal after Norwood palliation [4]. Li and colleagues showed that oxygen delivery was related to systemic vascular resistance and hemoglobin, not Qp alone [5]. Charpie and Photiadis reported that maximum oxygen delivery may occur at Qp:Qs less than 1 [7,8]. The present model reproduces this principle by showing that oxygen delivery can improve despite lower saturation when systemic flow is preserved or increased.

The Monte Carlo analysis strengthens this interpretation by demonstrating that the direction of drug effects remained stable despite uncertainty in baseline flow, baseline Qp:Qs, and resistance changes. This supports the concept that the clinical effect of pulmonary vasodilation depends strongly on starting physiology. In pulmonary undercirculation, pulmonary vasodilation may improve oxygenation and oxygen delivery if systemic flow is preserved. In balanced or over-circulated physiology, further reduction in pulmonary resistance may divert flow away from the systemic circulation and reduce oxygen delivery.

The advanced sensitivity analyses refine this interpretation. When total ventricular output is allowed to rise with afterload reduction, the oxygen-delivery penalty of pulmonary vasodilation may be attenuated or reversed if the increase in total flow is sufficient and if shunt/conduit resistance limits pulmonary runoff. Therefore, the model should not be read as a drug-specific prediction that pulmonary vasodilators are uniformly harmful. Rather, it identifies the physiologic conditions under which pulmonary vasodilation becomes risky: low shunt/conduit resistance, marked pulmonary selectivity, limited ventricular-flow reserve, and an already balanced or over-circulated baseline state.

The dose–response simulations further emphasize that modeled vasoactive effects should not be interpreted as binary drug responses. Across increasing modeled drug-effect fractions, nicardipine produced the greatest improvement in oxygen delivery, consistent with the benefit of systemic afterload reduction when systemic flow is preserved or increased. Milrinone, epoprostenol, and sildenafil showed smaller positive effects under the advanced assumptions, whereas inhaled nitric oxide remained slightly unfavorable. These findings suggest that the clinical effect of vasoactive therapy in Norwood physiology depends on the degree of resistance change, ventricular flow reserve, and the balance between native pulmonary and shunt/conduit resistance.

The findings also reinforce the importance of systemic venous saturation and other perfusion markers. Hoffman and colleagues showed that lower systemic venous oxygen saturation after the Norwood procedure was associated with abnormal childhood neurodevelopmental outcomes [9]. Dhillon and colleagues reported that routine hemodynamic parameters did not accurately reflect oxygen transport after Norwood palliation, except for systemic venous oxygen saturation [10]. Thus, oxygen saturation should be interpreted alongside systemic venous saturation, lactate, perfusion pressure, urine output, near-infrared spectroscopy, and clinical signs of end-organ perfusion.

Hemoglobin concentration is also a critical determinant of oxygen transport after Norwood palliation. In this model, hemoglobin was held constant to isolate the effects of flow redistribution and vascular resistance changes. This assumption should not be interpreted as minimizing the clinical importance of anemia. A patient may have an acceptable arterial oxygen saturation but impaired systemic oxygen delivery if hemoglobin concentration is low, because oxygen delivery depends on systemic blood flow, arterial oxygen saturation, and oxygen-carrying capacity.

Milrinone requires separate interpretation. In this model, milrinone was represented only by changes in systemic and native pulmonary vascular resistance. Clinically, milrinone also improves myocardial relaxation and contractility, which may be important in postoperative or preoperative single-ventricle patients [15,16]. Therefore, the milrinone results should be interpreted as a vascular-resistance simulation rather than a complete pharmacologic prediction. Under the assumptions of preserved ventricular function and balanced baseline Qp:Qs, milrinone produced a near-neutral effect on oxygen delivery, suggesting that its benefit may be limited when used primarily as a vasodilator in a Norwood circulation without impaired ventricular performance.

Computational modeling may be useful in Norwood physiology because flow distribution is difficult to infer from bedside saturation alone. Prior studies have used computational modeling and Doppler- or catheterization-based approaches to estimate postoperative flow distribution and oxygen delivery after Norwood palliation [11,12,13,25,26,27]. The present model is intended as a conceptual and educational tool to demonstrate how resistance changes can alter Qp:Qs and oxygen delivery.

Clinical Correlate: In bedside postoperative Norwood management, these simulations support interpreting vasoactive therapy in the context of systemic oxygen delivery rather than oxygen saturation alone. Pulmonary vasodilators may be appropriate when pulmonary blood flow is limited but should be used cautiously when there is concern for balanced or excessive pulmonary blood flow and systemic runoff. Conversely, systemic afterload reduction may improve systemic flow when ventricular function and perfusion pressure allow. In practice, pharmacologic decisions should remain individualized and should integrate systemic venous saturation, lactate, perfusion pressure, urine output, near-infrared spectroscopy, echocardiographic assessment, hemoglobin concentration, and estimates of Qp:Qs when available.

## 5. Limitations

This analysis is based on a simplified lumped-parameter model and should be interpreted as hypothesis-generating. Although the code includes an afterload–responsive total-flow sensitivity option, this remains a first-order approximation and is not a full time-varying elastance or pressure–volume loop model. The model therefore does not fully capture Frank-Starling recruitment, ventricular–arterial coupling, beat-to-beat contractility, atrioventricular valve regurgitation, coronary perfusion, respiratory mechanics, or rapidly changing postoperative physiology.

Baseline systemic resistance, native pulmonary resistance, shunt/conduit resistance, ventricular compliance, afterload sensitivity, and nonlinear shunt fraction were selected as physiologically plausible simulation assumptions rather than patient-specific measured values. The nonlinear shunt formulation improves on the purely linear Ohmic assumption, but true BT and Sano shunt flow may still be affected by geometry, viscosity, inertance, branch pulmonary artery stenosis, respiratory variation, and pulsatility.

Drug effects were modeled through changes in systemic and native pulmonary vascular resistance, with additional dose–response scaling in the revised analysis. Although the direction of each modeled resistance change was based on the published pharmacologic and hemodynamic literature, the exact dose–response curves and percent changes in resistance remain modeling assumptions and should not be interpreted as validated drug-specific effects in postoperative Norwood patients. This is particularly important for milrinone, which has inotropic and lusitropic effects that were not represented mechanistically.

The oxygen delivery index was simplified as Qs × mixed saturation. True systemic oxygen delivery requires systemic flow, hemoglobin concentration, arterial oxygen saturation, and dissolved oxygen. However, because hemoglobin and oxygen-carrying capacity were held constant, the index was useful for comparing directional changes across drug conditions.

The Monte Carlo analysis tested directional robustness rather than patient-specific probability distributions. The uncertainty ranges for baseline flow, baseline Qp:Qs, resistance changes, and effective pulmonary pathway resistance were selected as plausible sensitivity assumptions and were not derived from patient-level pharmacodynamic data. Clinical validation is required using postoperative Norwood data, including systemic venous oxygen saturation, lactate, near-infrared spectroscopy, echocardiographic flow estimates, catheterization-derived Qp:Qs, measured oxygen consumption, and high-fidelity physiologic monitoring.

The model also does not include pulsatile dynamics or diastolic pressure decay. The absence of pulsatile dynamics in the present model may lead to underestimation of the negative impact of vasodilator-induced diastolic pressure drops on coronary perfusion. This is particularly relevant in BT shunt physiology, where diastolic pulmonary runoff may compromise coronary perfusion and contribute to myocardial ischemia.

A formal Sobol global sensitivity analysis was not performed. However, the added Rp/R-shunt heatmap directly interrogates the most clinically relevant two-parameter resistance relationship and can serve as a foundation for future global sensitivity analysis.

## 6. Conclusions

In this computational Norwood circulation model, oxygen delivery was primarily determined by systemic flow, ventricular flow reserve, native pulmonary vascular resistance, and shunt/conduit resistance. In the primary fixed-flow analysis, nicardipine increased systemic flow and improved oxygen delivery, whereas selective pulmonary vasodilator conditions could increase modeled oxygen saturation while reducing systemic flow. In the extended sensitivity analyses, these effects were modified by afterload–responsive changes in total ventricular output and by nonlinear shunt behavior.

These findings reinforce that oxygen saturation alone is insufficient to assess postoperative Norwood physiology. An intervention that increases pulmonary blood flow may improve arterial saturation while reducing systemic oxygen delivery. Clinical interpretation should therefore integrate Qs, Qp:Qs, systemic venous saturation, perfusion pressure, lactate, and oxygen delivery rather than relying on saturation alone.

## Figures and Tables

**Table 1:** Hemodynamic and oxygen delivery effects of drug conditions from a balanced Norwood baseline.

Drug	ΔRs	ΔRp	Qs	Qp	Qp:Qs	Sm	Ssv	Oxygen Delivery Index	Change in Oxygen Delivery Index
Baseline	—	—	1.00	1.00	1.00	0.800	0.610	0.800	Reference
Nicardipine	−30.0%	−5.0%	1.15	0.85	0.74	0.769	0.601	0.882	+10.3%
Milrinone	−18.0%	−22.5%	0.97	1.03	1.06	0.803	0.610	0.782	−2.2%
Sildenafil	−10.0%	−30.0%	0.88	1.12	1.29	0.819	0.604	0.718	−10.2%
Inhaled nitric oxide	0.0%	−45.0%	0.71	1.29	1.82	0.843	0.575	0.598	−25.2%
Epoprostenol	−15.0%	−40.0%	0.83	1.17	1.42	0.828	0.598	0.685	−14.4%

Drug effects were modeled as percent changes in systemic vascular resistance and native pulmonary vascular resistance. Shunt/conduit resistance was held constant. Oxygen delivery index was calculated as Qs × Sm. Abbreviations: ΔRs, percent change in systemic vascular resistance; ΔRp, percent change in native pulmonary vascular resistance; Qs, systemic flow; Qp, pulmonary flow; Qp:Qs, pulmonary-to-systemic flow ratio; Sm, mixed saturation; Ssv, systemic venous saturation.

**Table 2:** Monte Carlo sensitivity analysis of drug effects from baseline Qp:Qs 1:1 and total flow 2 L/min.

Drug	Qs Mean, 95% UI	Qp Mean, 95% UI	Qp:Qs Mean, 95% UI	Sm Mean, 95% UI	Ssv Mean, 95% UI	Oxygen Delivery Index Mean, 95% UI	Oxygen Delivery Index Change	Probability Oxygen Delivery Index Decreases
Nicardipine	1.15 (0.91–1.42)	0.85 (0.64–1.07)	0.74 (0.56–0.94)	0.762 (0.694–0.812)	0.595 (0.500–0.665)	0.881 (0.654–1.119)	+9.9% (+4.5 to +16.0)	0.0%
Milrinone	0.98 (0.75–1.22)	1.03 (0.79–1.27)	1.06 (0.80–1.36)	0.802 (0.750–0.841)	0.604 (0.515–0.671)	0.783 (0.582–0.997)	−2.2% (−9.5 to +4.2)	73.4%
Sildenafil	0.88 (0.67–1.10)	1.12 (0.87–1.39)	1.29 (0.97–1.65)	0.818 (0.772–0.853)	0.599 (0.506–0.667)	0.720 (0.536–0.917)	−10.2% (−18.1 to −2.9)	99.8%
Inhaled nitric oxide	0.71 (0.53–0.92)	1.29 (1.01–1.58)	1.83 (1.36–2.41)	0.841 (0.802–0.870)	0.569 (0.463–0.648)	0.599 (0.439–0.780)	−25.2% (−34.9 to −16.2)	100.0%
Epoprostenol	0.83 (0.63–1.05)	1.17 (0.91–1.45)	1.42 (1.06–1.87)	0.825 (0.781–0.859)	0.593 (0.498–0.663)	0.687 (0.508–0.882)	−14.3% (−23.8 to −5.8)	100.0%

Monte Carlo simulation used 20,000 iterations. Baseline total right ventricular flow was varied around 2.0 L/min, baseline Qp:Qs was varied around 1:1, and drug-related changes in systemic and native pulmonary vascular resistance were varied around the modeled values. Shunt/conduit resistance was held constant. Oxygen delivery index was calculated as Qs × Sm. Values are reported as mean with 95% uncertainty interval. Abbreviations: Qs, systemic flow; Qp, pulmonary flow; Qp:Qs, pulmonary-to-systemic flow ratio; Sm, mixed saturation; Ssv, systemic venous saturation; UI, uncertainty interval; 95% uncertainty interval, 2.5th to 97.5th percentile range from the Monte Carlo simulation.

**Table 3:** Advanced sensitivity analysis using afterload–responsive total flow and nonlinear shunt resistance.

Drug	Qtotal	Qs	Qp	Qp:Qs	Oxygen Delivery Change
Nicardipine	2.145	1.237	0.908	0.735	+20.7%
Milrinone	2.098	1.113	0.985	0.884	+10.9%
Sildenafil	2.071	1.046	1.025	0.980	+5.2%
Inhaled nitric oxide	2.053	0.969	1.084	1.118	−1.3%
Epoprostenol	2.104	1.076	1.028	0.955	+8.3%

## Data Availability

The original contributions presented in this study are included in the article. Further inquiries can be directed to the corresponding author.
